# Design Principles of Catalytic Materials for CO_2_ Hydrogenation to Methanol

**DOI:** 10.1002/adma.202409322

**Published:** 2024-09-19

**Authors:** Thaylan Pinheiro Araújo, Sharon Mitchell, Javier Pérez‐Ramírez

**Affiliations:** ^1^ Institute for Chemical and Bioengineering Department of Chemistry and Applied Biosciences ETH Zurich Vladimir‐Prelog‐Weg 1 Zurich 8093 Switzerland

**Keywords:** catalyst nanostructures, CO_2_ hydrogenation, methanol synthesis, operando characterization, reducible metal oxides

## Abstract

Heterogeneous catalysts are essential for thermocatalytic CO_2_ hydrogenation to methanol, a key route for sustainable production of this vital platform chemical and energy carrier. The primary catalyst families studied include copper‐based, indium oxide‐based, and mixed zinc–zirconium oxides‐based materials. Despite significant progress in their design, research is often compartmentalized, lacking a holistic overview needed to surpass current performance limits. This perspective introduces generalized design principles for catalytic materials in CO_2_‐to‐methanol conversion, illustrating how complex architectures with improved functionality can be assembled from simple components (e.g., active phases, supports, and promoters). After reviewing basic concepts in CO_2_‐based methanol synthesis, engineering principles are explored, building in complexity from single to binary and ternary systems. As active nanostructures are complex and strongly depend on their reaction environment, recent progress in operando characterization techniques and machine learning approaches is examined. Finally, common design rules centered around symbiotic interfaces integrating acid–base and redox functions and their role in performance optimization are identified, pinpointing important future directions in catalyst design for CO_2_ hydrogenation to methanol.

## Graphical abstract

Heterogeneous catalysts are the key for sustainable methanol production via CO_2_ hydrogenation. By overviewing recent advances in the most promising catalyst families, this perspective identifies generalized design rules centered around symbiotic interfaces integrating acid–base and redox functions and their role in performance optimization, proposing future research directions.

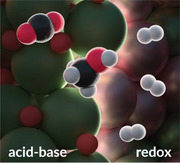

## 1 Introduction

**Figure 1:**
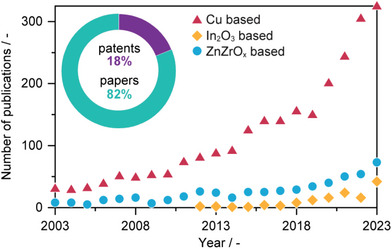
Total number of articles and patents between 2003 and 2023 on heterogeneously catalyzed CO_2_ hydrogenation to methanol. Yearly trends in the total number of publications based on the most investigated classes of catalysts. Data was retrieved from Web of Science.

With a global production of ≈110 Mt per year, methanol is crucial for manufacturing numerous other chemicals and is a prospective fuel for the maritime shipping industry.^[^ ^1^, ^2^, ^3^ ^]^ Driven by the latter, the demand for methanol will increase dramatically, with estimates reaching ≈360–500 Mt per year by 2050.^[^ ^3^, ^4^ ^]^ However, current methanol production relies heavily on fossil feedstocks, necessitating a shift toward carbon‐neutral routes to effectively meet this growth while addressing the critical issue of achieving net‐zero emissions in the chemical industry and transportation sector.^[^ ^4^, ^5^ ^]^ In this context, carbon dioxide hydrogenation using renewable H_2_ and captured CO_2_ offers an effective framework for sustainable methanol production (CO_2_ + 3H_2_ ⇌ CH_3_OH + H_2_O), as demonstrated by systems analysis at the planetary level.^[^ ^1^, ^2^, ^5^ ^]^ Alongside cost‐effective supply chains for raw materials, catalytic materials are central to realizing such transformation at the required scale. However, developing efficient heterogeneous catalysts is challenging due to the inert nature of CO_2_ and the variety of products that can form upon its hydrogenation.^[^ ^1^, ^6^, ^7^ ^]^ This topic has stimulated extensive research to identify promising catalytic materials and rationalize their performance, as evidenced by the surge in related papers and patents over the past 20 years (**Figure** **1**).

Three primary families of catalysts have been reported for CO_2_ hydrogenation to methanol: i) copper‐based, ii) indium oxide (In_2_O_3_)‐based, and iii) mixed zinc–zirconium oxides (ZnZrO*_x_*)‐based materials (Figure 1).^[^ ^6^, ^8^, ^9^, ^10^, ^11^, ^12^ ^]^ Copper‐based systems, notably Cu–ZnO–Al_2_O_3_, emerged in 1966 for large‐scale methanol production from syngas (CO + 2H_2_ ⇌ CH_3_OH).^[^ ^10^, ^13^ ^]^ The success of these catalysts sparked interest in translating them for CO_2_‐to‐methanol conversion assuming the requirement of analogous reaction paths. Consequently, Cu–ZnO–Al_2_O_3_ and related formulations remain the subject of extensive study (Figure 1b).^[^ ^8^, ^10^, ^13^ ^]^ Cu‐based materials are characterized by their high activity but limited methanol selectivity and stability. Comparatively, reducible oxide catalysts such as In_2_O_3_ and ZnZrO*_x_* were only discovered in 2015 and 2017, respectively.^[^ ^14^, ^15^, ^16^ ^]^ The high selectivity and stability exhibited by reducible oxide catalysts have attracted excitement from academic and industrial arenas (Figure 1). However, improvements in H_2_ activation and overall activity are still key targets.^[^ ^9^, ^12^, ^17^, ^18^ ^]^ Despite the progress within each family, catalyst design efforts have often been compartmentalized, lacking a more holistic approach, which is also mirrored in several outstanding reviews on the topic.^[^ ^6^, ^8^, ^9^, ^10^, ^13^, ^18^, ^19^, ^20^ ^]^ A broader and comprehensive perspective on the design of catalytic materials for CO_2_ hydrogenation to methanol is essential for advancing catalyst development, thus facilitating the discovery of novel materials with improved performance.

This perspective outlines keydesign principles for heterogeneous catalysts in CO_2_ hydrogenation to methanol, using recent examples to illustrate how simple components can be combined to construct complex architectures with enhanced functionality. After introducing underlying thermodynamic and mechanistic concepts, we summarize the engineering principles for catalyst architectures, moving from single to binary and ternary materials. Given the dependence of catalyst nanostructures on reaction conditions, we also examine the current challenges in characterizing active sites. This holistic analysis helps identify common design rules, highlighting the central role of symbiotic catalytic interfaces in performance optimization. Lastly, we explore emerging and future directions in catalyst design for CO_2_ hydrogenation.

## 2 CO_2_ Hydrogenation to Methanol

### 2.1 Thermodynamic Considerations

Compared to the large‐scale production of methanol from synthesis gas (also known as syngas, a mixture of H_2_, CO, and CO_2_, Equation 1), CO_2_‐based methanol synthesis requires an additional H_2_ to remove an oxygen from CO_2_, forming water as a byproduct (Equation 2).^[^ ^7^, ^21^ ^]^ In addition, CO_2_ hydrogenation to methanol competes with the reverse water‐gas shift (RWGS, Equation (3)), which primarily produces carbon monoxide (CO).^[^ ^6^, ^22^ ^]^ While this CO can subsequently be hydrogenated to methanol (Equation 1), this depends on the catalyst used and reaction kinetics.

(1) $$\mathrm{CO}+2H_{2}\;⇌\;CH_{3}\mathrm{OH},\Delta H_{298\;K}=\;-90.6\;\mathrm{kJ}\;\mathrm{mo}l^{-1}$$

(2) $$CO_{2}\;+\;3H_{2}\;⇌\;CH_{3}\mathrm{OH}+\;H_{2}O,\Delta H_{298\;K}\;=\;-49.5\;\mathrm{kJ}\;\mathrm{mo}l^{-1}$$

(3) $$CO_{2}\;+\;H_{2}\;⇌\;\mathrm{CO}+\;H_{2}O,\Delta H_{298\;K}=\;41.2\;\mathrm{kJ}\;\mathrm{mo}l^{-1}$$

**Figure 2:**
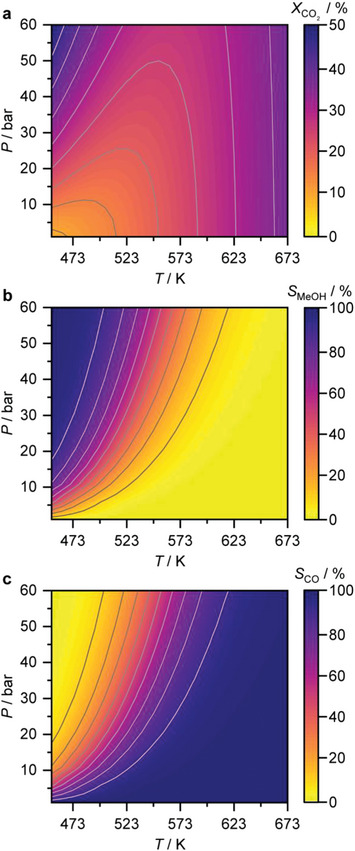
Thermodynamic equilibrium in terms of a) CO_2_ conversion, *X*_CO2_; b) methanol; and c) CO selectivity; *S*_i_ (*I* = MeOH or CO) during CO_2_ hydrogenation as a function of reaction temperature (*T*) and pressure (*P*). Results shown in (a–c) are attained via simulations using Aspen Plus. Numbers and lines colored in white or gray correspond to the *X*_CO2_ or *S*_i_ at a given *T* and *P*.

From a thermodynamic standpoint, Le Châtelier's principle indicates that methanol synthesis from CO_2_ and CO is generally more favored at low temperatures and high pressures (**Figure** **2**a,b).^[^ ^7^, ^22^ ^]^ This preference is due to the exothermic nature of these reactions (Equations (1) and (2)) and the reduced number of molecules as the reaction proceeds forward.^[^ ^7^, ^22^ ^]^ In contrast, the RWGS is more favored at high temperatures but is virtually insensitive to pressure changes as it is endothermic and does not change the total number of molecules (Equation (3) and Figure 2c).^[^ ^22^ ^]^ While these thermodynamic considerations suggest the RWGS could be suppressed in favor of methanol synthesis by tuning the temperature, the chemically inert nature of CO_2_ pushes reaction temperatures up. Methanol synthesis is generally operated at 493–573 K and 50–100 bar, conditions that unfortunately also favor CO formation, reducing the methanol yield, which is already heavily limited by the thermodynamic equilibrium.^[^ ^7^, ^23^, ^24^ ^]^ Although advances in reaction condition optimization and reactor designs, covered in depth in previous reviews,^[^ ^7^, ^18^, ^22^ ^]^ offer important ways to mitigate these limitations, a key element to improving methanol productivity lies in designing highly active, selective, and stable catalysts.

### 2.2 Reaction Mechanisms

**Figure 3:**
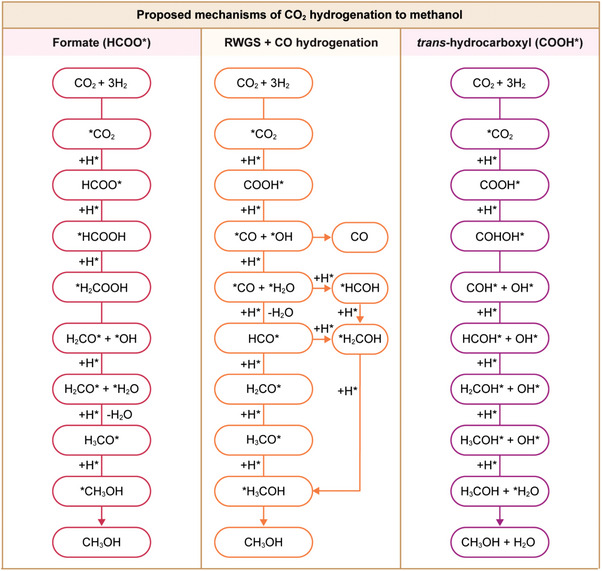
Reaction pathways and key intermediates proposed for CO_2_ hydrogenation to methanol. RWGS stands for reverse water gas‐shift reaction, whereas * indicates adsorbed species. Adapted with permission.^[^ ^26^ ^]^ Copyright 2017, American Chemical Society.

Various reaction mechanisms have been proposed for catalyzed CO_2_ hydrogenation to methanol.^[^ ^7^, ^24^, ^25^, ^26^ ^]^ Here, we recap key steps of the most reported pathways supported by experimental and computational approaches (**Figure** **3**), while detailed descriptions for relevant catalytic materials are covered in Section 3. Two intermediates, carboxyl (COOH*, where * refers to adsorbed species) and formate (HCOO*), lie at the center of debates on the main reaction routes.^[^ ^6^, ^7^, ^10^, ^27^, ^28^ ^]^ Most studies favor the HCOO* pathway in which the formate intermediate is formed by the reaction of CO_2_ with preadsorbed surface atomic H (Figure 3).^[^ ^29^, ^30^ ^]^ According to these studies, the subsequent hydrogenation of HCOO* generates dioxomethylene (H_2_COO*), with further reaction steps leading to formaldehyde (H_2_CO*), methoxy (CH_3_O*), and finally CH_3_OH. Recently, a revised HCOO* pathway put forward that formic acid (HCOOH*) is preferentially formed instead of H_2_COO*, which is hydrogenated to H_2_COOH*, and then splits to generate H_2_CO* and *OH, with a final hydrogenation step leading to methanol via the CH_3_O* intermediate (Figure 3).^[^ ^31^ ^]^ This revised mechanism has gained support over the years as more studies using density functional theory (DFT) have shown that activation barriers to form H_2_COO* from HCOO* are markedly higher than for HCOOH*.^[^ ^32^, ^33^ ^]^

Another frequently reported mechanism involves the RWGS reaction followed by CO hydrogenation to methanol, which begins with CO_2_ conversion to COOH* (Figure 3).^[^ ^7^, ^34^, ^35^, ^36^ ^]^ This pathway has been suggested to effectively explain the formation of CO byproduct as COOH* can decompose into CO* and *OH. The former can then desorb as a product or be further hydrogenated to methanol via formyl (HCO*), formaldehyde (H_2_CO*), and H_3_CO* intermediates. Although the CO hydrogenation pathway is widely accepted, and CO formation is primarily reported to occur through the decomposition of the COOH* intermediate, direct CO_2_ decomposition (i.e., CO_2_* → CO* + O*) has also been proposed as a potential source of CO*.^[^ ^7^, ^26^ ^]^ Overall, the RWGS + CO hydrogenation mechanism is considered more favorable than the HCOO* pathway to forming methanol over some catalysts as it proceeds faster.^[^ ^6^ ^]^ However, the preference for the RWGS + CO hydrogenation mechanism depends on the CO adsorption strength on a given catalytic surface.^[^ ^26^, ^35^ ^]^ If CO binds weakly, the catalyst is expected to be more selective toward CO due to the facile desorption of *CO. Conversely, if CO binds strongly, its hydrogenation is likely more favorable than desorption, promoting higher methanol selectivity.

Although formate species are often observed experimentally during CO_2_ hydrogenation to methanol, some studies claim they might not be the active intermediates, but rather stable spectators.^[^ ^7^, ^37^, ^38^, ^39^ ^]^ Instead, the less commonly discussed *trans*‐COOH mechanism has been proposed, wherein COOH* rather than HCOO* species is the first hydrogenation intermediate (Figure 3). Unlike the RWGS + CO hydrogenation mechanism, this pathway does not rely on the dissociation of COOH* to form methanol. Instead, CO_2_ reaction with H atoms provided by H_2_O initially generates COOH* that is subsequently hydrogenated to dihydrocarbene (COHOH*) and dissociates to yield hydromethylidyne (COH*). Further hydrogenation steps lead to methanol formation via hydroxymethylene (HCOH*) and hydromethyl (H_2_COH*) intermediates. Support for the *trans*‐COOH mechanism stems from some studies suggesting that direct hydrogenation of formate to methanol under dry H_2_ does not yield methanol, whereas adding water results in the desired product.^[^ ^38^, ^40^ ^]^ In addition, theoretical studies indicate that methanol formation through the HCOO* pathway is unfeasible due to the high barriers for hydrogenating formate and H_2_COO* species, compared to the lower barriers of the *trans*‐COOH route enabled by H_2_O.^[^ ^37^ ^]^ Despite these arguments, strong experimental evidence supporting the *trans*‐COOH mechanism is still lacking as COOH* species are difficult to detect using conventional in situ vibrational spectroscopies, thus necessitating transient operando techniques.^[^ ^6^, ^26^ ^]^

### 2.3 H_2_ Activation

**Figure 4:**
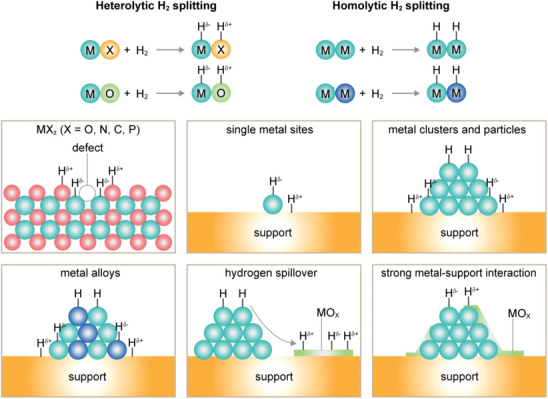
Scheme of heterolytic and homolytic hydrogen (H_2_) splitting processes over key catalyst architectures for CO_2_ hydrogenation to methanol. M and MO*_x_* stand for metal and metal oxide, respectively. SMSI indicates strong metal–support interactions. Adapted with permission: Mechanism and structures,^[^ ^41^ ^]^ is licensed under CC‐BY 4.0; hydrogen spillover,^[^ ^42^ ^]^ copyright 2017, American Chemical Society.

Catalytic materials that can efficiently activate H_2_ are critical for methanol synthesis from CO_2_, particularly when using renewable H_2_, which contributes significantly to the higher costs associated with sustainable methanol production over fossil‐based routes.^[^ ^1^, ^3^, ^18^ ^]^ This section briefly reviews the main modes of H_2_ activation and their implications for relevant active architectures in CO_2_‐to‐methanol synthesis (**Figure** **4**). H_2_ dissociation occurs through two primary mechanisms: heterolytic and homolytic pathways. Using heterogeneous catalysts, the former often involves a metal center (*M*) bound to a nonmetal atom (e.g., *X* = O, N, S, C, or P) acting as a Lewis acid–base pair to dissociat H_2_ into a proton‐hydride pair (*X*–H*^δ^* ^+^ and *M*H*^δ^* ^−^, respectively).^[^ ^17^, ^41^ ^]^ In contrast, homolytic H_2_ dissociation typically occurs on metal surfaces, where the nature of the *M*─H bond (hydridic [*M*─H*^δ^* ^−^] or covalent [*M*‐H]) depends on the relative electronegativity of the metal. Late transition metals, commonly used in CO_2_ hydrogenation to methanol, predominantly exhibit *M*H bonds due to their similar electronegativity to hydrogen.^[^ ^41^, ^42^ ^]^

Heterogenous catalysts can sometimes exhibit these distinct modes simultaneously, resulting in the coexistence of *X*–H*^δ^* ^+^, *M*–H*^δ^* ^−^, and *M*–H species.^[^ ^42^, ^43^ ^]^ This occurs in catalytic materials containing multiple types of active sites that can independently split H_2_ in a homolytic or heterolytic manner. In addition, hydrogen atoms formed via homolytic H_2_ dissociation are highly mobile and can migrate across the metal surface to metal–support interfaces, a process known as hydrogen spillover.^[^ ^17^, ^42^, ^43^ ^]^ This process typically creates *X*–H*^δ^* ^+^ and *M*–H*^δ^* ^−^ pairs. Interestingly, this spillover phenomenon is not limited to the immediate support surface but can extend to a second accepting surface (e.g., reducible oxides) of different composition.^[^ ^42^ ^]^ However, this extended spillover is less efficient than having a secondary phase, especially *M*O*_x_*, adjacent to the metal surface formed due to strong metal–support interactions (SMSI).^[^ ^44^ ^]^

## 3 Engineering Catalyst Architectures

**Figure 5:**
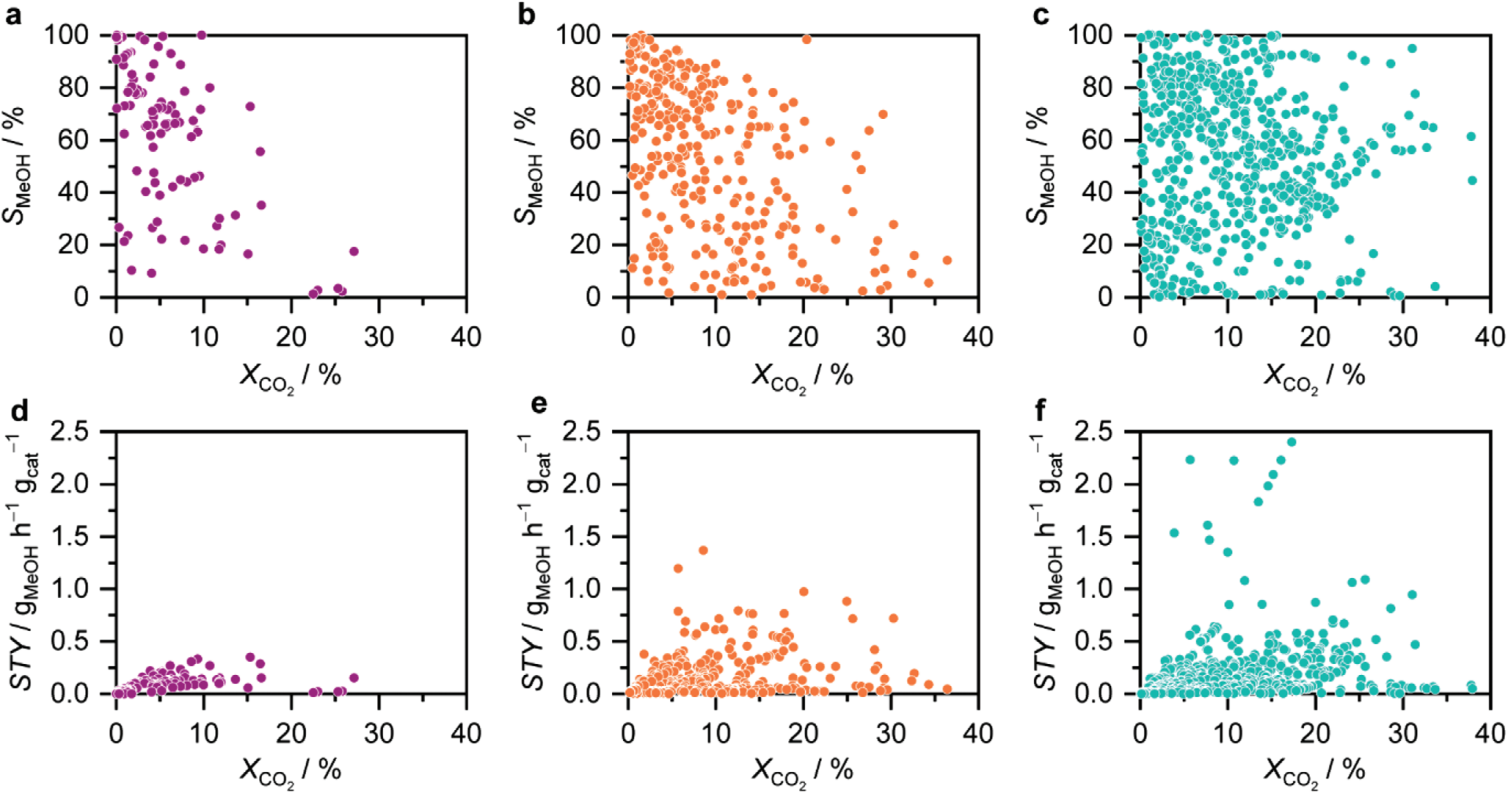
a–c) Methanol selectivity, *S*_MeOH_; and d–f) methanol space‐time yield, *STY*, as a function of CO_2_ conversion, *X*_CO2_ over single (a,d), binary (b,e), and ternary catalytic systems developed in the last decades (c,f). The literature dataset includes *S*_MeOH_, methanol STY, and *X*_CO2_ reported for catalysts evaluated during CO_2_ hydrogenation at distinct reaction conditions.

Building on this thermodynamic and kinetic basis, this section focuses on how catalysts have been developed to achieve active, selective, and stable CO_2_ hydrogenation performance. Previous reviews have tackled single classes of catalyst materials, such as Cu‐, In_2_O_3_‐, or ZnZrO*_x_*‐based catalysts. This compartmentalized approach lacks a holistic view of catalyst design. In each case, development has progressed similarly: identifying a catalytically active phase and then making the design more complex to improve functionality, much like assembling components in a toy building set. Here, we adopt this approach, where simple components—active phases, supports, and promoters—are assembled into complex architectures for enhanced performance. We categorize catalysts into single, binary, and ternary catalytic materials. Single catalytic materials (SCM) combine a (post‐)transition metal and a non‐metal atom to form a single phase (e.g., metal oxide, carbide, nitride, or phosphide). Binary catalytic materials (BCMs) include two (post‐)transition metals and one or more non‐metal atoms, forming either a single phase (solid solution) or distinct interfacial phases. Ternary catalytic materials (TCMs) incorporate a third (post‐)transition metal with one or more non‐metal atoms, creating distinct interfacial phases. Analyzing the performance of key catalytic materials reported over the last 2 decades, we observe that most studies focus on TCM, followed by BCM, with SCMs being significantly less reported (**Figure** **5**a–c). SCMs generally exhibit high methanol selectivity (*S*_MeOH_) but limited CO_2_ conversion (*X*_CO2_), leading to a low methanol space‐time yield (*STY*). Conversely, BCM and TCM typically achieve higher *X*_CO2_ while maintaining similar *S*_MeOH_ levels as SCM, resulting in improved methanol STY. To understand these trends and identify similarities and differences in design approaches, we further explore the current understanding of synthesis–property–function relationships for each class of catalytic materials. It is noteworthy to highlight that the temperature ranges at which these catalysts achieve maximum STY can vary significantly due to the diverse compositional and structural space covered by binary and ternary systems compared to single‐component materials. Further, it is also essential to consider other reaction parameters such as pressure, CO_2_/H_2_ ratio, and gas‐hourly space velocity. These factors significantly influence methanol STY and *X*_CO2_ and are crucial for developing accurate guidelines for optimizing CO_2_‐to‐methanol and other related applications, such as methanol‐to‐olefins conversion.

### 3.1 Single Catalytic Materials

**Figure 6:**
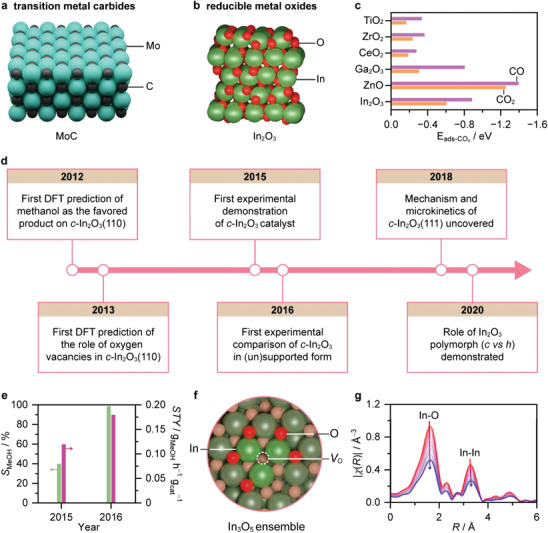
Progress in the design of single catalytic materials (SCM). Scheme of a) molybdenum carbide (MoC) and b) indium oxide (In_2_O_3_) structures, which represent key classes of SCM. c) CO_2_ and CO adsorption energies (E_ads−CO_ *_x_*, CO*_x_* = CO_2_ or CO) on distinct metal oxide surfaces predicted by DFT simulations. Adapted with permission.^[^ ^9^ ^]^ Copyright 2021, American Chemical Society. d) Timeline showing landmarks driving catalyst design in In_2_O_3_‐catalyzed CO_2_ hydrogenation to methanol. e) Methanol selectivity, *S*_MeOH_, and space‐time yield, *STY*, during CO_2_ conversion for the first In_2_O_3_ catalysts, reported in 2015 (reaction conditions: *T* = 603 K, *P* = 4 MPa, GHSV = 15 000 cm^3^ h^−1^ g_cat_^−1^, and H_2_/CO_2_ = 3) and 2016 (reaction conditions: *T* = 573 K, *P* = 5 MPa, GHSV = 21 000 cm^3^ h^−1^ g_cat_^−1^, and H_2_/CO_2_ = 4), respectively.^[^ ^14^, ^15^ ^]^ f) Representation of identified active catalytic ensemble for In_2_O_3_ catalysts. Adapted with permission.^[^ ^56^ ^]^ Copyright 2018, Elsevier. g) Fourier‐transform of the *k* ^2^‐weighted operando In *K*‐edge EXAFS for In_2_O_3_ catalyst during CO_2_ hydrogenation (reaction conditions: *T* = 573 K, *P* = 2 MPa, GHSV = 1 000 000 cm^3^ h^−1^ g_cat_^−1^, and H_2_/CO_2_ = 3). The arrows indicate the direction of changes with time‐on‐stream (*TOS* = 140 min). Adapted with permission.^[^ ^59^ ^]^ Copyright 2019, American Chemical Society.

Single catalytic materials (SCM) are notable for their ability to activate CO_2_ and H_2_ to produce methanol without the need for promoters or supports. Prominent examples include transition metal carbides^[^ ^27^, ^28^, ^45^ ^]^ (TMC, e.g., molybdenum carbide (MoC), **Figure** **6**a) and reducible metal oxides^[^ ^9^, ^12^ ^]^ (RMO, e.g., indium oxide (In_2_O_3_), Figure 6b). Research on TMC, which has a long history, primarily involves theoretical studies using methods such as DFT, often combined with experimental work using model catalysts synthesized under oxidant‐free atmospheres.^[^ ^27^, ^28^ ^]^ Although studies on powders, particularly MoC, often confirm the activity observed in model catalysts, their methanol selectivity, and notably, stability are limited.^[^ ^28^, ^45^, ^46^, ^47^ ^]^ Stability losses primarily occur due to the contact with CO_2_ and H_2_O, which tend to oxidize the carbide phase, leading to catalyst deactivation. Much more robust than TMC, RMO, particularly in powder form, has recently garnered significant attention as SCM for CO_2_‐to‐methanol.^[^ ^12^ ^]^ Among several examples of RMO such as gallium oxide (Ga_2_O_3_), cerium oxide (CeO_2_), titanium oxide (TiO_2_), zirconium oxide (ZrO_2_), and zinc oxide (ZnO), In_2_O_3_ has emerged as the most prominent due to its superior activity and methanol selectivity.^[^ ^9^ ^]^ The performance of these oxides can be correlated with their CO_2_ and CO adsorption energies (Figure 6c).^[^ ^48^, ^49^, ^50^, ^51^, ^52^, ^53^, ^54^ ^]^ In_2_O_3_ offers an optimal balance; its CO_2_ adsorption energy is neither so weak that it does not interact with the surface and react nor so strong that the surface coverage becomes so high it limits the conversion. Similarly, its CO adsorption energy is neither so weak that CO desorbs easily, increasing CO selectivity, nor so strong that it poisons the surface. Given its relevance, we will focus on catalyst design for In_2_O_3_, using it as a primary example of SCM.

The potential of In_2_O_3_ as an active and selective phase for CO_2_ hydrogenation to methanol was first proposed in 2012 by a DFT study on the cubic (*c*‐)In_2_O_3_(110) surface (Figure 6d).^[^ ^55^ ^]^ Although this seminal work did not explicitly indicate that methanol is formed over this particular surface, it demonstrated that *c*‐In_2_O_3_(110) can activate CO_2_ and H_2_ while suppressing CO formation, thereby favoring formate species, which is a common intermediate for methanol synthesis (see Section 2.2). Further key findings included that CO_2_ is initially adsorbed and activated over a surface O atom to form carbonate species and that H_2_ activation occured either heterolytically (forming In–H*^δ^* ^−^ and O–H*^δ^* ^+^) or homolytically (forming two O–H*^δ^* ^+^), but only the former favored formate formation upon hydrogenation of carbonate species. Although this study provided valuable insights into the first steps of the hydrogenation of CO_2_ on *c*‐In_2_O_3_(110), it assumed a perfect surface, overlooking the propensity of In_2_O_3_ to reduce to In_2_O_3‐_ *_x_*, generating surface oxygen vacancies (*V*_O_).^[^ ^9^ ^]^ A subsequent DFT study on the *c*‐In_2_O_3_(110) surface in 2013 addressed the creation, stability, and role of *V*_O_ in methanol synthesis (Figure 6d),^[^ ^48^ ^]^ finding that they enhance CO_2_ activation and hydrogenation and stabilize key methanol synthesis intermediates such as formate, which is both thermodynamically and kinetically favored. In addition, methanol formation was reported to replenish *V*_O_, while H_2_ helped generate these sites, creating a cycle vital to catalyzing methanol production.

Two experimental works published in 2015 and 2016 were the first to confirm theoretical predictions using In_2_O_3_ catalysts (Figure 6d,e).^[^ ^14^, ^15^ ^]^ The first study focused on commercial In_2_O_3_, which exhibited moderate *S*_MeOH_ and methanol STY (≈40% and 0.12 g_MeOH_ h^−1^ g_cat_^−1^, respectively, (Figure 6e)) even at high temperatures (i.e., 603 K), which typically favor the RWGS reaction.^[^ ^14^ ^]^ Further corroborating these findings, the second study evaluated a self‐made In_2_O_3_ catalyst, achieving a higher *S*_MEOH_ and methanol STY (≈99% and 0.18 g_MeOH_ h^−1^ g_cat_^−1^, respectively, Figure 6e) at a slightly lower temperature but higher pressure (i.e., 573 K and 5 MPa, respectively).^[^ ^15^ ^]^ These results confirm the ability of In_2_O_3_ to suppress CO formation and underscore the significant influence of the reaction conditions on the catalytic performance of In_2_O_3_.^[^ ^15^ ^]^ Preliminary ex situ characterization using H_2_ temperature programmed reduction and electron paramagnetic spectroscopy suggested that the unique performance of In_2_O_3_ was most likely due to the presence of oxygen vacancies. Interestingly, the same study also demonstrated for the first time the impact of supports on the performance of In_2_O_3_ (see Section 3.2).

Combined computational and experimental studies have since deepened understanding of the catalytic properties of In_2_O_3_.^[^ ^56^, ^57^, ^58^ ^]^ Notably, a comprehensive kinetic investigation of In_2_O_3_^[^ ^57^ ^]^ showed that the apparent activation energy (*E*_a_,_app_) for methanol synthesis (*E*_app_,_MeOH_ = 103 kJ mol^−1^) is lower than for the RWGS reaction (*E*_a_,_app_,_CO_ = 118 kJ mol^−1^), explaining the superior methanol selectivity of In_2_O_3_ (Figure 6d).^[^ ^56^ ^]^ Focusing on the predominant *c*‐In_2_O_3_(111) surface, this study identified In_3_O_5_ as the active ensemble (Figure 6f), likely formed via the abstraction of surface oxygen atoms due to the anisotropic nature of these atoms in the oxide lattice. The catalytic ensemble activated CO_2_ and heterolytically split H_2_, following the most energetically favored path (formate mechanism), involving three consecutive additions of hydrides and protons to form methanol. Crucially, hydrogen splitting was uncovered as the rate‐determining step. Research on the role of the In_2_O_3_ polymorph is another example where theoretical and experimental work were intertwined (Figure 6d).^[^ ^57^, ^58^ ^]^ Hexagonal (*h*)‐In_2_O_3_ exhibited higher activity and methanol selectivity compared to *c*‐In_2_O_3_, attributed to oxygen vacancies on the *h*‐In_2_O_3_(104) facet that stabilized key intermediates in methanol formation.^[^ ^57^ ^]^ However, *h*‐In_2_O_3_ could transform into *c*‐In_2_O_3_ during CO_2_ hydrogenation, and the latter was thermodynamically more stable, making it a focal point of theoretical and experimental studies.^[^ ^9^, ^57^, ^58^ ^]^

Most studies agree that oxygen vacancies are the active sites or precursors of them in In_2_O_3_ catalysts.^[^ ^9^, ^56^ ^]^ However, as revealed by an operando study combining several characterization techniques including extended X‐ray absoprtion fine structure (EXAFS) via X‐ray absorption spectroscopy (XAS) (Figure 6g), an increased density of oxygen vacancies can become problematic.^[^ ^59^ ^]^ Under reaction conditions, the high reducibility of In_2_O_3_ can make its structure highly dynamic, resulting in reductive amorphization and a continuous interconversion between amorphous/molten indium (In^0^) and crystalline In_2_O_3−_ *_x_* domains, ultimately causing catalyst deactivation.^[^ ^59^ ^]^

### 3.2 Binary Catalytic Materials

**Figure 7:**
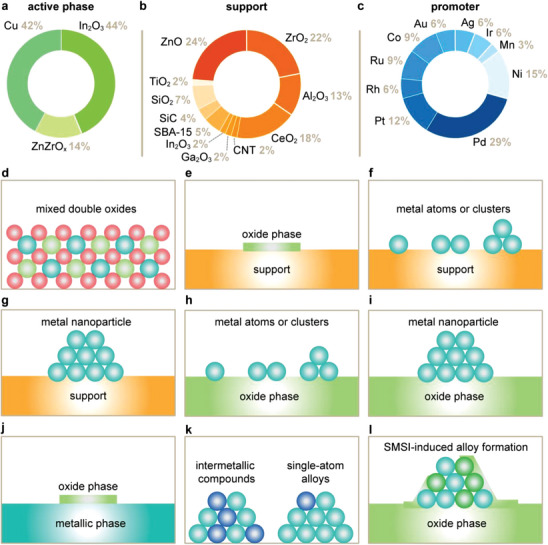
Most common a) active phase, b) supports, and c) promoters reported for binary catalytic materials (BCM). The literature dataset used for the statistical analyses shown in (a–c) is available online.^[^ ^124^ ^]^ d–l) Schemes of the most prominent active architectures reported for BCM.

Binary catalytic materials (BCMs) offer a promising platform for enhancing CO_2_ hydrogenation performance. By integrating metal promoters or supports with active phases (**Figure** **7**a–c), BCMs can potentially address the inherent limitations of SCMs such as In_2_O_3_. These strategies also allow for the synergistic activation of distinct phases that are otherwise considerably less active (e.g., Cu and ZnO) or virtually inactive (ZrO_2_) when used individually, forming new and more effective catalysts. Supports like ZrO_x_, ZnO, and CeO_2_ play crucial roles in maximizing the dispersion of active phases, stabilizing intermediates, and ensuring the longevity of catalytic systems (Figure 7b). Transition metals such as Pd and Ni are widely favored as promoters (Figure 7c) for enhancing performance by improving hydrogen activation, especially over In_2_O_3_ (Figure 7a). While seemingly straightforward, this classification overlooks the intricate interactions among components, which result in dynamic structural and compositional changes that give rise to novel interfacial phenomena, thereby generating a diverse array of potential active catalytic architectures (Figure 7d–l). Here, we examine the synergy within binary catalytic systems, seeking to elucidate how these interactions can be leveraged to enhance catalytic performance.

**Figure 8:**
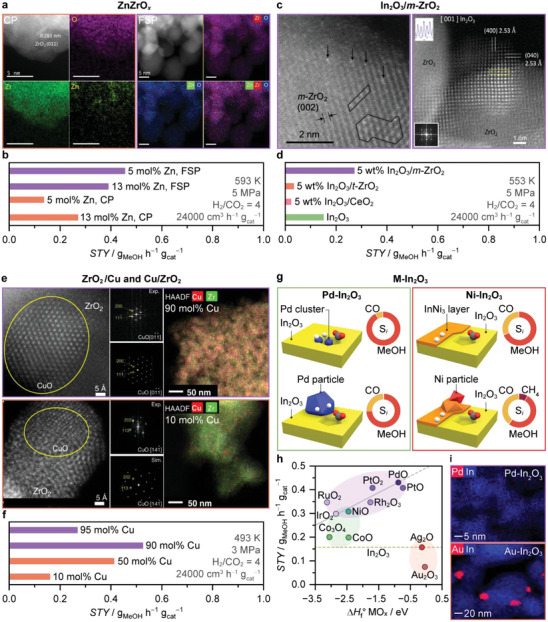
Selected examples of binary catalytic materials (BCM). a,c,e) Visualization and corresponding methanol space‐time yield, b,d,f) STY of ZnZrO*_x_*, In_2_O_3_/*m*‐ZrO_2_, and Cu/ZrO_2_ catalysts, with other BCM shown for reference. Reproduced with permission: panels a,b coprecipitation^[^ ^16^ ^]^ copyright 2017, AAAS and FSP^[^ ^63^ ^]^ is licensed under CC BY‐NC‐ND 4.0; panels c,d left^[^ ^66^ ^]^ copyright 2020, American Chemical Society and right^[^ ^67^ ^]^ copyright 2022, American Chemical Society; panels e,f^[^ ^69^ ^]^ are licensed under CC BY 4.0. g) Schemes of identified active architectures for Pd‐ and Ni‐In_2_O_3_ catalysts and corresponding product selectivity (*S*_i_ = MeOH, CO, or CH_4_). Structures are adapted from^[^ ^72^, ^73^ ^]^ licensed under CC BY 4.0. h) Relationship between methanol STY during CO_2_ hydrogenation over *M*‐In_2_O_3_ catalysts versus the experimental standard enthalpy of formation of the most stable metal oxide, *M*O*_x_* for each metal promoter (Δ_f_H *M*O*_x_*) normalized by the number of metal atoms in the stoichiometric unit. The STY obtained over undoped In_2_O_3_ (yellow dashed line) is marked for comparison. Reaction conditions: *T* = 553 K, *P* = 5 MPa, *GHSV* = 24 000 cm^3^ h^−1^g_cat_^−1^, and H_2_/CO_2_ = 4. i) Energy‐dispersive X‐ray spectroscopy (EDX) maps of Pd and Au–In_2_O_3_ after activation in CO_2_ hydrogenation for 2 h. Reaction conditions: *T* = 553 K, *P* = 5 MPa, GHSV = 24 000 cm^3^ h^−1^ g_cat_^−1^, and H_2_/CO_2_ = 4. Data in panels h,i^[^ ^83^ ^]^ is licensed under CC BY‐NC 4.0.

ZnZrO*_x_* (**Figure** **8**a,b) represents a special case of BCMs as neither ZnO nor ZrO_2_ alone are promising SCMs nor do they strictly function as a support or metal promoter in this context. However, their combination results in an active system. Since they were introduced in 2017, ZnZrO*_x_* systems have garnered significant attention due to their high methanol selectivity and stability, even in the presence of impurities that often cause catalyst deactivation.^[^ ^16^, ^60^, ^61^, ^62^, ^63^ ^]^ Following its discovery, most studies on ZnZrO*_x_* focused on coprecipitation (CP) rather than impregnation as the preferred synthesis method as initial work suggested that forming a solid solution phase (Figures 7d and 8a) was key to maximizing active Zn–O–Zr motifs necessary to obtaining high methanol STY.^[^ ^16^, ^60^, ^61^ ^]^ However, recent reports have significantly deepened our understanding of synthesis–structure–performance relationships for ZnZrO*_x_* systems. ZnZrO*_x_* prepared by flame spray pyrolysis (FSP) achieved superior catalytic performance compared to CP (Figure 8b). Using lower Zn contents (5 mol% Zn), FSP maximized catalyst surface area and atomic dispersion of Zn in the surface lattice of ZrO_2_, enhancing oxygen vacancy formation without the need for a bulk solid solution.^[^ ^63^ ^]^ These vacancies, along with surrounding Zn and Zr–O atoms, created active ensembles favoring methanol formation through the formate mechanism. Alternatively, a more recent study revisited impregnation methods, uncovering that this approach also produces active catalysts that share several features with FSP counterparts.^[^ ^62^ ^]^ Notably, a speciation switch from low to high nuclearity was observed around the optimum Zn content (5 mol%). At this point, the ZrO_2_ surface was saturated with catalytically active isolated Zn sites, and facile oxygen vacancy generation in neighboring sites formed the active Zn–*V*_O_–Zr ensemble under reaction conditions.^[^ ^63^ ^]^ Importantly, the specific surface area (*S*_BET_) of ZrO_2_, overlooked in previous studies, emerged as a key performance descriptor. Indeed, maximizing *S*_BET_ enhanced the ZrO_2_ capability to stabilize a greater amount of dispersed Zn species. Remarkably, the optimal catalyst architecture and performance were retained when transitioning from powder to technical forms. Despite these achievements, there is still room for performance improvement of ZnZrO*_x_*, particularly by enhancing its hydrogen splitting ability, which can potentially be addressed by introducing a metal promoter, as discussed in Section 3.3.

In_2_O_3_/monoclinic (*m*‐)ZrO_2_ is a prime example of BCMs where the integration of a specific support is critical to enhancing the performance of a SCM (Figures 7e and 8d,c). First reported in 2016, this BCM exhibited remarkable stability for 1000 h on stream and higher methanol STY (Figure 8d), while optimizing the utilization of In_2_O_3_ and preserving its characteristic high methanol selectivity.^[^ ^15^ ^]^ Building upon these findings, subsequent studies delved deeper into the role of ZrO_2_, crucial for prospective industrial applications of In_2_O_3_.^[^ ^64^, ^65^, ^66^, ^67^ ^]^ A 2020 study examined geometric, electronic, and interfacial phenomena through distinct synthesis methods, revealing that CP produced solid solution catalysts (Figure 7d), composed mostly of tetragonal (*t*‐) ZrO_2_, which exhibited an inferior performance compared to In_2_O_3_/*m‐*ZrO_2_ obtained by wet impregnation (WI). This ruled out strong electronic interactions as the main reason for the enhanced activity observed in ZrO_2_‐supported In_2_O_3_ catalysts.^[^ ^66^ ^]^ Further, In_2_O_3_/*m‐*ZrO_2_ exhibited ≈ten times higher methanol STY compared to other catalysts where In_2_O_3_ was supported on *t*‐ZrO_2_ or CeO_2_ by WI (Figure 8d). Kinetic investigations revealed that In_2_O_3_/*t*‐ZrO_2_ and In_2_O_3_/CeO_2_ displayed similar apparent activation energy (*E*_a,app_) as In_2_O_3_ alone, which was higher than that of In_2_O_3_/*m‐*ZrO_2_ (*E*_a,app_ = 106 vs 89 kJ mol^−1^). In addition, In_2_O_3_/*m‐*ZrO_2_ showed lower reaction orders for H_2_ (*n*_H2_ = 0.3 vs 0.5) and CO_2_ (*n*_CO2_ = 0.0 vs −0.1) compared to In_2_O_3_, indicating facilitated hydrogen activation and a less inhibiting role of CO_2_ in methanol synthesis,^[^ ^66^ ^]^ which likely followed the formate mechanism.^[^ ^67^, ^68^ ^]^ The superior performance of In_2_O_3_/*m‐*ZrO_2_ is attributed to several intertwined factors, including: i) high dispersion of In_2_O_3_ forming monolayers or low nuclearity species (Figure 8c), ii) higher density of oxygen vacancies on the epitaxially supported In_2_O_3_ due to lattice strain from the specific mismatch with m‐ZrO_2_, and iii) high CO_2_ adsorption capacity and activation ability of *m*‐ZrO_2_ to activate CO_2_.^[^ ^9^, ^66^ ^]^ While most studies confirm the superior and unique properties of *m‐*ZrO_2_ as a support for In_2_O_3_, the exact identity of the active architecture remains up for debate.^[^ ^64^, ^65^, ^66^, ^67^ ^]^ Besides the examples discussed above,^[^ ^66^ ^]^ some studies claim that In_2_O_3_ undergoes reconstruction under reaction conditions, inducing dynamic surface segregation and redispersion of In_2_O_3_ into the lattice of *m*‐ZrO_2_ or even the formation of permanent surface solid solutions, creating active In–*V*o–Zr ensembles.^[^ ^64^, ^67^ ^]^ However, no study has yet thoroughly investigated the dynamic behavior of In_2_O_3_ and its impact on performance.

The intricate interplay between copper and various supports establishes this combination as pivotal among BCMs, offering a vast canvas for crafting active architectures, yet simultaneously poses challenges in establishing synthesis–structure–performance relationships. A compelling illustration of this phenomenon is observed when combining Cu and ZrO_2_, where distinct copper speciation has been documented to exert a significant impact on CO_2_ hydrogenation to methanol (Figures 7f,g,j and 8e,f).^[^ ^19^, ^69^, ^70^, ^71^ ^]^ For instance, Cu/ZrO_2_ catalysts prepared using the CP method accumulate undercoordinated cationic Cu_1_–O_3_ species dynamically on the ZrO_2_ surface (Figure 7f).^[^ ^70^ ^]^ These species form stable isolated centers during CO_2_ hydrogenation at 453 K, achieving 100% selectivity to methanol. This specific architecture facilitates hydrogen dissociation aided by nearby oxygen atoms, activating CO_2_ to produce HCOO*. This pathway is exclusively viable on the Cu_1_–O_3_ sites, contrasting with small copper clusters or nanoparticles (Figure 7g) that promote the RWGS route. Large copper particles exhibit minimal CO_2_ activation at applied conditions. In another report, an inverse ZrO_2_/Cu catalyst comprising 10 mol% of ZrO_2_ supported on metallic Cu particles (Figures 7j and 8e) exhibited a threefold increase in methanol STY compared to traditional zirconia‐supported Cu (Cu/ZrO_2_, Figures 7g and 8e) catalysts at 493 K (Figure 8f).^[^ ^69^ ^]^ Detailed characterization revealed that the ZrO_2_/Cu systems consisted of islands of partially reduced amorphous ZrO_2_ (≈1–2 nm) supported over metallic Cu particles. These islands exhibited high activity in CO_2_ activation, facilitating faster formation and consumption of formate and methoxy intermediates compared to the Cu/ZrO_2_ configuration. Further, the formation of a highly reactive HCOO* species on the metallic Cu component of the inverse ZrO_2_/Cu catalyst contributes significantly to its superior performance. Overall, these findings underscore the importance of considering various copper speciations when developing effective catalysts for CO_2_ hydrogenation to methanol.

Promoting an active oxide phase with metals represents a crucial strategy for developing highly active BCMs (Figure 7 h,i,k,l). As hydrogen activation remains the main challenge, the promotion of In_2_O_3_ with hydrogenation metals (Figure 7c) has become a key research focus within BCMs.^[^ ^9^ ^]^ However, identifying optimal metal promoter architectures is not a trivial task due to the complex interplay with product distribution. Hence, understanding the intricate synthesis–structure–performance relationships of *M*‐In_2_O_3_ catalysts is essential for efficiently promoting In_2_O_3_. In this context, Pd and Ni‐promoted systems, prepared by CP and WI, respectively, are textbook examples of how to precisely tailor metal speciation for enhancing the hydrogen splitting ability of In_2_O_3_, while attaining high stability and untouched selectivity and, consequently, high methanol productivity (Figure 8g).^[^ ^72^, ^73^ ^]^ Contrasting Pd nanoparticles, metallic Pd clusters containing ≈three atoms stabilized on the In_2_O_3_ surface are key to efficiently splitting H_2_ while suppressing CO formation (Figures 7h,i and 8g).^[^ ^73^ ^]^ For Ni, InNi_3_ alloys in the form of layers supported on In_2_O_3_ are formed upon reaction and are critical to suppress both CO and, especially, methane formation, which is particularly favored over bare metallic Ni particles.^[^ ^72^ ^]^ Despite various other metal promoters being explored in the literature,^[^ ^14^, ^72^, ^73^, ^74^, ^75^, ^76^, ^77^, ^78^, ^79^, ^80^, ^81^, ^82^ ^]^ rationalization of the reported results is challenging due to the distinct synthesis protocols, metal amounts, and testing conditions applied. Aiming to standardize the evaluation, a study introduced FSP as a platform to systematically preparing *M*‐In_2_O_3_ systems via a single‐step synthesis, providing a detailed analysis on the speciation and promotional effects of nine *M*‐In_2_O_3_ (*M* = Au, Ag, Co, Ni, Ir, Ru, Rh, Pt, and Pd) catalysts (Figure 8h,i).^[^ ^83^ ^]^

A key descriptor for *M*‐In_2_O_3_ was attained by correlating the methanol STY over *M*‐In_2_O_3_ for each promoter with the standard formation enthalpy (Δ_f_H) of its oxide *M*O*_x_* (Figure 8h). As a general rule, the most active promoters are those more metallic (less exothermic Δ_f_H) and atomically dispersed (i.e., Ir, Ru, Rh, Pt, and Pd, Figure 8i) as they more efficiently promote homolytic H_2_ splitting. However, if the promoter has a very poor oxygen affinity, it does not stabilize by the In_3_O_5_ ensemble; and therefore, will nucleate into metallic nanoclusters and nanoparticles (Figure 8i) under synthesis conditions (i.e., Ag and Au). In contrast, if these metals are stabilized as adatoms on In_2_O_3_ surface (In_3_ *M*O*_x_*), they should be highly active. Finally, promoters with high oxygen affinity (i.e., Co) tend to incorporate into the bulk or to form mixed metal oxides.

### 3.3 Ternary Catalytic Materials

**Figure 9:**
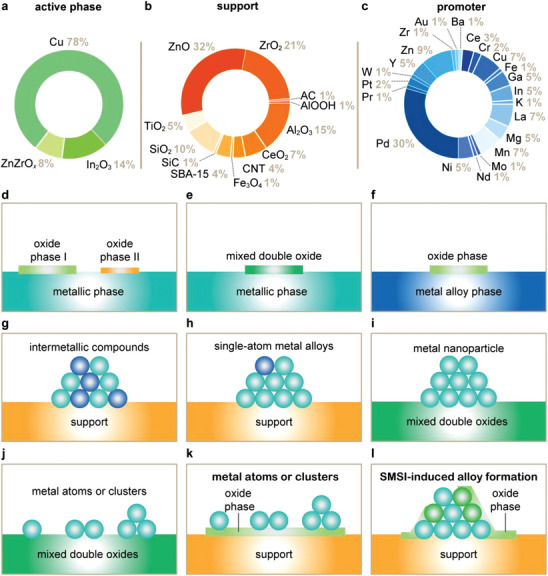
Most common a) active phases, b) supports, and c) promoters reported for ternary catalytic materials (TCM). The literature dataset used for the statistical analyses shown in (a–c) can be found in the Supporting Information. d–l) Schemes of the most prominent active architectures reported for TCM.

Ternary catalytic materials (TCMs) present a complex yet fascinating landscape for tailoring CO_2_ hydrogenation performance. The component phases of TCMs can be broadly categorized into active phases, promoters, and supports (**Figure** **9**a–c). Cu, In₂O₃, and ZnZrO_x_ are generally considered active phases due to their intrinsic capability to facilitate methanol synthesis independently. Numerous promoters have been introduced to enhance the performance further by modifying the active sites or providing additional reaction pathways, with Pd and Zn emerging as the most common. Supports like ZnO, ZrO_x_, and Al₂O₃ are recognized to play a vital role in stabilizing the overall structure, ensuring the longevity and durability of the catalytic system. While this classification offers a straightforward framework, it is important to acknowledge the intricate interactions between these components. These interactions can induce significant structural and compositional changes, often leading to novel interfacial phenomena that can alter the nature of the active species. The multitude of potential architectures and the resultant effects underscore the complexity and potential of ternary systems (Figure 9d–l). Here, we explore the synergy within ternary catalytic systems, aiming to understand how these interactions can be harnessed to optimize catalytic performance.

Among the most important ternary systems studied include the catalysts industrially applied for methanol synthesis from CO and CO_2_ (Cu–ZnO–Al_2_O_3_), as well as metal‐promoted ZnZrO*_x_* and supported In_2_O_3_‐based systems. Current research focuses on understanding the role and interplay of each of the three component phases within each family and determining the reaction mechanism.^[^ ^13^ ^]^ In the case of the most widely studied Cu–ZnO–Al_2_O_3_, copper is typically present in high molar percentages (e.g., >50 mol%) in the form of metallic nanoparticles (a structure analogous to Figure 9d) and is normally thought of as the main active phase. Studies have linked ensuring a high surface area of Cu nanoparticles and the presence of numerous defects such as stacking faults^[^ ^84^ ^]^ with enhanced catalytic activity. These defects are stable under industrial conditions and improve the interaction with ZnO, promoting dynamic changes primarily at the Cu surface rather than in the bulk. The interaction of Cu with adsorbed oxygen species, possibly originating from water dissociation or the ZnO interface, leads to partial oxidation of Cu atoms, forming electron‐depleted Cu*^δ^* ^+^ species that are thought to be vital for adsorbing and activating CO_2_. Moreover, oxygen species at the CuZnO interface reinforce adhesion, facilitating the hydrogenation process.

**Figure 10:**
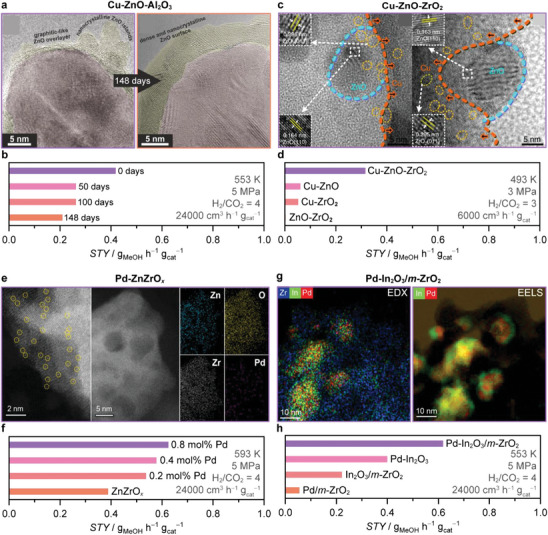
Selected examples of ternary catalytic materials (TCM). Visualization and corresponding methanol space‐time yield; STY of a,b) Cu–ZnO–Al_2_O_3_, c,d) Cu–ZnO–ZrO_2_, e,f) Pd–ZnZrO*_x_*, and g,h) Pd‐In_2_O_3_/*m*‐ZrO_2_ catalysts, with other TCM shown as reference. Reproduced or adapted with permission: Cu–ZnO–Al_2_O_3_^[^ ^90^ ^]^ copyright 2016, Wiley‐VCH; Cu–ZnO–ZrO_2_^[^ ^96^ ^]^ is licensed under CC BY 4.0; Pd–ZnZrO*_x_* ^[^ ^97^ ^]^ copyright 2022, Elsevier; Pd‐In_2_O_3_/m‐ZrO_2_^[^ ^44^ ^]^ is licensed under CC BY 4.0.

ZnO, on the other hand, has been proposed to act synergistically with Cu through several mechanisms. There is a consensus that it acts as a physical spacer to enhance Cu dispersion.^[^ ^32^ ^]^ It is also generally accepted that it stabilizes active Cu^+^ sites, promoting their formation. Experimental evidence has also supported the formation of Cu–Zn alloys that create active sites for CO_2_ activation.^[^ ^85^, ^86^ ^]^ Other studies have revealed the formation of metastable graphitic‐like ZnO overlayers over Cu during the reaction,^[^ ^87^ ^]^ linked to a strong metal‐support interaction (SMSI), leading to a type of inverse catalyst structure. Most recent studies agree that Zn species on metallic Cu^0^ particles have a positively charged nature,^[^ ^88^, ^89^ ^]^ with the proportion of CuZnO*_x_* interfacial sites recently proposed as a descriptor for catalyst activity.^[^ ^88^ ^]^ The ZnO phase is known to be highly dynamic and studies over long‐term tests have shown that performance losses result from the restructuring and sintering of the Cu particles (**Figure** **10**a,b).^[^ ^90^ ^]^

Less is known about the role of Al_2_O_3_ (10 mol%), which is widely recognized for its function as a structural promoter, increasing the surface area available for active Cu sites and ensuring the structural integrity and stability of the catalyst during operation. Microscopy studies have shown that the Al_2_O_3_ phase is also intimately mixed and finely decorates the partially ZnO‐covered Cu particles, likely forming mixed Al–Zn oxides and possibly other phases.^[^ ^91^ ^]^ While its direct electronic effects are less explored, Al^0^ doping of ZnO or Cu has been linked to promotional effects.^[^ ^92^, ^93^ ^]^ Comparatively, other oxides such as ZrO_2_^[^ ^94^ ^]^ and CeO_2_^[^ ^95^ ^]^ have shown varying activity and selectivity, with ZrO_2_ sometimes outperforming Al_2_O_3_ due to its basic sites, which could enhance CO_2_ adsorption and subsequent hydrogenation steps.^[^ ^54^ ^]^ To further understand this system, a recent study prepared a 3D‐ordered mesoporous material out of copper acting as a support for ZnO and ZrO_2_ particles of different sizes (Figure 10c).^[^ ^96^ ^]^ The Cu–ZnO–ZrO_2_ catalyst substantially outperformed BCM analogs (Figure 10d). Compared to the BCM where methanol productivity is determined by the metal–metal oxide interface, the TCM exhibited higher methanol yield even though the Cu surface area was lower, which has been attributed to the key role of the ZnO–ZrO_2_ interface. Increased oxygen vacancy formation was proposed to facilitate CO_2_ adsorption based on X‐ray photoelectron spectroscopy (XPS) results, while insitu diffuse reflectance infrared Fourier transform spectroscopy (DRIFTS) studies suggested that the ZnO–ZrO_2_ interface was crucial for transforming carbonate to formate.^[^ ^96^ ^]^

The next TCM family to emerge for CO_2_ hydrogenation was metal‐promoted ZnZrO*_x_* catalysts. Efforts in their development have demonstrated significant performance enhancements by incorporating various metals, most prominently palladium (0.11 wt%)^[^ ^97^, ^98^, ^99^ ^]^ and copper (0.5 mol%),^[^ ^100^ ^]^ although a broad range has been studied.^[^ ^101^ ^]^ Control over the promoter speciation, ensuring high dispersion, is paramount for maximizing improvements in STY from these metals. Atomic dispersions of promoters have been successfully achieved through different synthetic approaches including coprecipitation (Figure 10e) and flame spray pyrolysis (FSP). Herein, the inclusion of palladium promoters has increased methanol STYs by up to 50% (Figure 10f). The presence of the metal promoter can impact the atomic structure of the ZnZrO*_x_* phase, for example, analysis of a reduced Pd/ZnZr catalyst showed that it transformed from a ternary solid solution into a binary phase where Pd^2+^ segregated from the *t*‐ZrO_2_ lattice forming a PdZn alloy with ZnO.^[^ ^98^ ^]^

The metal promoters in ZnZrO_x_ systems can play multiple roles. Generally, it is expected that the presence of palladium will facilitate H_2_ activation and subsequent formate formation, significantly reducing the activation energy and improving methanol production rates. Nonetheless, other studies suggest that the main impact of Pd incorporation is to increase surface oxygen vacancy formation, thus boosting the adsorption and activation of CO_2_ and accelerating methanol production.^[^ ^97^, ^98^ ^]^ Similarly, copper promotion via FSP generates Zn‐rich CuZn clusters that enhance oxygen vacancy formation and improve catalytic performance, doubling methanol productivity compared to unpromoted ZnZrO*_x_*.^[^ ^101^ ^]^ These CuZn clusters, integrated with zinc ensembles on the ZrO_2_ surface, facilitate rapid hydrogenation of formate intermediates and suppress CO formation, thereby enhancing methanol selectivity and stability. Considering the lower price and environmental footprint compared to Pd, Cu stands out as the most promising promoter to date. The exact role of metal promoters likely depends significantly on their speciation and distribution over the catalyst and is still not fully understood. Nonetheless, the efficient transfer of H atoms, either through the proximity of H_2_ and CO_2_ activating sites or through engineering the delivery,^[^ ^99^ ^]^ appears critical.

In the case of In_2_O_3_‐based systems, ternary Pd–In_2_O_3_–ZrO_2_ catalysts have shown significant potential for CO_2_ hydrogenation to methanol, leveraging the synergistic effects of palladium promotion and zirconia supports.^[^ ^44^, ^102^, ^103^, ^104^ ^]^ The successful integration of these components had been achieved by scalable methods such as wet impregnation and FSP, exhibiting enhanced methanol productivity and stability compared to their binary counterparts. The use of FSP resulted in low‐nuclearity palladium species associated with In_2_O_3_ monolayers highly dispersed on the ZrO_2_ carrier, facilitating a transformation of the surface structure from tetragonal to monoclinic‐like upon reaction.^[^ ^102^ ^]^ This structural evolution and the creation of oxygen vacancies significantly boosted catalytic performance. Using mixed monoclinic and tetragonal ZrO_2_ phases as supports was subsequently shown to yield superior reactivity due to the increased concentration of oxygen vacancies and medium basic sites, essential for activating H_2_ and CO_2_.^[^ ^103^ ^]^

For Pd‐In_2_O_3_–ZrO_2_ catalysts synthesized via impregnation, rapid surface restructuring occurs during CO_2_ hydrogenation, generating a distinct active architecture to FSP.^[^ ^44^ ^]^ This process involves the formation of InPd*_x_* alloy particles decorated with InO*_x_* layers, which stabilize palladium nanoparticles and prevent sintering (Figure 10g). The interaction between metal and metal oxide phases is crucial, as evidenced by operando X‐ray diffraction (XRD) and XAS. These techniques reveal that the InO*_x_* species migrate onto the Pd surface, forming an alloy that enhances H_2_ activation and promotes methanol formation while suppressing CO production (Figure 10h). Notably the mechanism of reconstruction and the impact of reaction environments remain unresolved. Further, the relationship among the nanostructure, CO_2_ adsorption and activation, and H_2_ splitting and transfer has yet to be fully established.

Overall, while SCMs offer a simpler design and are intrinsically highly selective for methanol, they often exhibit limitations such as low *X*_CO2_, insufficient H_2_ dissociation, and consequently, low methanol productivity. In addition, SCMs may underutilize critical materials such as In_2_O_3_. BCMs address several of these limitations by incorporating a support, a metal promoter, or a second phase, such as in mixed solid solutions. However, due to the limited components in BCM formulations, these systems generally enhance only one specific property of SCMs at a time. TCMs significantly increase the complexity of catalytic formulations by combining an active phase with a support and a metal promoter. Still, this complexity enables TCMs to design more diverse and multifaceted catalysts that integrate multiple advantageous attributes of BCMs, effectively addressing several limitations of SCMs simultaneously.

## 4 Challenges in Characterizing Active Site Architectures

Characterizing active site structures is fundamental to catalyst design as it directly influences our ability to optimize and stabilize catalytic systems. The overview of advances across distinct catalyst families in Section 3 highlights the increasing complexity of catalytic formulations. Further, it emphasizes the importance of selecting appropriate synthetic approaches and supports to stabilize the optimal nanostructures of active phases and promoters. Precise characterization under realistic conditions is crucial for controlling these structures. In situ and operando techniques are indispensable in this effort, offering insights into the true working state of catalysts that static or ex situ measurements may not capture. As recent reviews have explored the in situ and operando study of CO_2_ hydrogenation catalysts, this section recaps key aspects and focuses on current challenges.^[^ ^105^, ^106^, ^107^ ^]^

**Figure 11:**
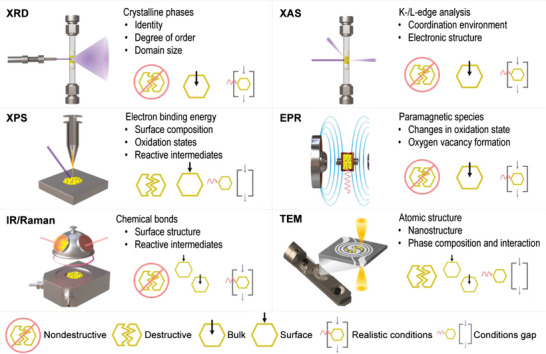
Summary of complementary in situ/operando experimental probes available to investigate chemical, electronic, and structural evolution of heterogeneous catalysts for CO_2_ hydrogenation to methanol. The applicability and limitations of each technique are highlighted. X‐ray diffraction, XRD; X‐ray absorption spectroscopy, XAS; near‐ambient pressure X‐ray photoelectron spectroscopy, NAP‐XPS; electron paramagnetic spectroscopy, EPR; diffuse reflectance infrared Fourier transform spectroscopy, DRIFTS.

Certain techniques are more amenable to in situ application and have been most applied for determining active structures of CO_2_ hydrogenation catalysts, particularly X‐ray‐based methods (such as XRD, XAS, and XPS), and other spectroscopic methods such as electron paramagnetic resonance (EPR), IR, and Raman (**Figure** **11**). Significant steps have also been taken to advance in situ transmission electron microscopy (TEM) in all its modalities. These methods have been invaluable in providing complementary information about different features of the catalytic system under investigation. However, each tool has specific limitations, including restricted accessibility, gaps between experimental designs and relevant operating conditions, and the complexity of correlating observed properties to the catalyst nanostructure.^[^ ^108^, ^109^ ^]^

**Figure 12:**
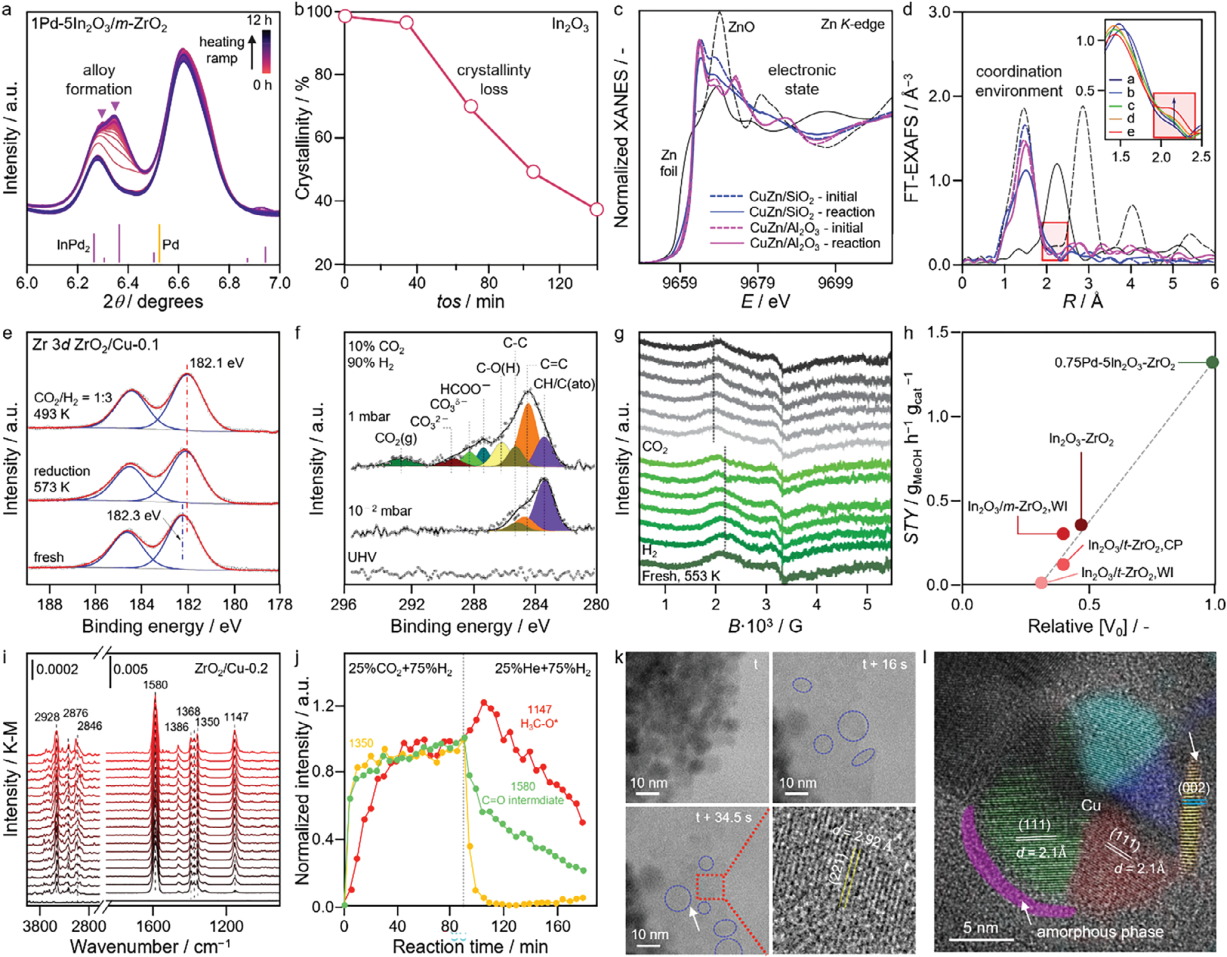
Selected examples of insights derived from in situ/operando investigations using the techniques summarized in Figure 11. a,b) XRD studies identifying the formation of In_2_Pd alloy or monitoring the crystallinity losses. c,d) XAS analysis showing the differences in electronic and geometric state of catalysts when analyzed ex situ or in situ. e,f) NAP‐XPS monitoring changes in oxidation state of catalyst components or the formation of reactive intermediates. g,h) EPR studies quantifying the extent of oxygen vacancy formation, identifying a correlation with the methanol STY. i,j) DRIFTS investigation of the formation of reactive intermediates. k,l) TEM visualization of the phase distribution, identifying amorphous or crystalline regions in In_2_O_3_ or the segregation of ZnO islands in Cu–Zn–Al systems. Panels adapted or reproduced with permission: panel a^[^ ^44^ ^]^ is licensed under CC BY 4.0; panels b,k^[^ ^59^, ^112^ ^]^ copyright 2019, American Chemical Society; panels c,d^[^ ^111^ ^]^ are licensed under CC BY 4.0; panels e,i,j^[^ ^69^ ^]^ are licensed under CC BY 4.0; panel f^[^ ^125^ ^]^ copyright 2018 Wiley‐VCH; panels g,h^[^ ^102^ ^]^ are licensed under CC BY 4.0; panel l^[^ ^91^ ^]^ is licensed under CC BY‐NC 4.0.

Operando XRD and XAS analysis of bulk structures have been widely used, often in combination, because they can be operated at high pressure (≥10 bar), which is the main challenge for in situ studies of CO_2_ hydrogenation. Operando XRD enables tracking phase changes and structural transformations during the catalytic process and is generally non‐destructive. For example, in the case of In_2_O_3_‐based systems, analysis of the SCM evidences its amorphization as the reaction proceeds, which is linked to catalyst deactivation (**Figure** **12**a).^[^ ^59^ ^]^ In the case of TCMs, it provides key insights into the nanostructure of the palladium promoter, identifying the formation of Pd–In alloys (Figure 12b).^[^ ^44^ ^]^ Comparatively, operando XAS offers detailed information on electronic states and atomic environments of component elements, primarily focusing on metals, depending on the specific implementation (e.g., EXAFS and XANES).^[^ ^110^ ^]^ Studies of single‐phase systems follow changes in the coordination environment such as decreasing In─O and In─In coordination numbers associated with the activation due to oxygen vacancy formation in In_2_O_3_.^[^ ^59^ ^]^ In ternary Cu/ZnO/Al_2_O_3_ catalysts, analysis of the X‐ray absorption near‐edge structure (XANES) spectra at the Cu *K*‐edge confirms the complete reduction of Cu species under reaction conditions.^[^ ^111^ ^]^ At the same time, inspection of the Zn *K*edge XANES spectra reveals the coexistence of reduced Zn species with ZnO (Figure 12c). Changes related to the partial reduction of Zn species can also be observed in the Zn K‐edge EXAFS spectra, which confirms the gradual formation of ZnZn or ZnCu bonds as the reaction proceeds (Figure 12d).

Key limitations of operando XRD and XAS studies are that they typically provide information on bulk structures, and it is often unclear how the insights gained reflect the properties of the active catalyst surfaces. Further, XRD is restricted to phases with a certain degree of crystalline order, while the structural insights derived from XAS represent averaged properties over the whole catalyst structure, and it is often challenging to discriminate polydisperse species present in low concentrations. In this regard, near ambient pressure (NAP‐)XPS extends the capability to study surface chemical properties and catalytic roles.^[^ ^112^, ^113^ ^]^ For instance, a study of model catalysts varying the coverage of indium on a copper foil showed that with low coverage, a small amount of In is completely oxidized upon exposure to CO_2_.^[^ ^112^ ^]^ Comparison of traditional binary Cu/ZrO_2_ and inverse ZrO_2_/Cu catalysts showed that the Zr species are partially reduced in the inverse system (Figure 12e), which is not observed in the traditional structure, indicating that highly dispersed ZrO_2_ domains over Cu particles contain a significant amount of O defects in reductive atmospheres. By studying the O1*s* and C1*s* regions, the potential of NAP‐XPS to detect key intermediates is also demonstrated (Figure 12f), identifying species consistent with a formate mechanism over a single‐phase Cu(111) surface, thus enhancing our understanding of the surface chemistry involved in CO_2_ hydrogenation.^[^ ^113^ ^]^ Nonetheless, although advances have enabled a shift away from ultra‐high vacuum conditions, the pressure gap to CO_2_ hydrogenation reaction conditions remains large and most studies have focused on model systems. Correlation with observations at higher pressures and developing approaches for studying multicomponent systems are required to further integrate NAP‐XPS approaches.

Another spectroscopic method that has recently emerged as a powerful approach for operando characterization is EPR. Operando EPR was recently exploited to probe oxygen vacancy formation in a ternary Pd–In_2_O_3_–ZrO_2_ catalyst. The distinctive broadening of the vacancy‐related signal observed under flowing H_2_ with time‐on‐stream showed that oxygen vacancy formation was augmented by H_2_ and not just thermally induced (Figure 12g).^[^ ^44^ ^]^ A correlation was demonstrated between the number of oxygen vacancies and the methanol space‐time yield independent of the catalyst preparation method, highlighting the key importance of these defects (Figure 12h). Subsequent analysis of the influence of Zn species on oxygen vacancy formation in ZnZrO*_x_* systems helped elucidate their formation mechanism, showing that zinc incorporation into the lattice of monoclinic zirconia creates these vacancies and peroxide species, with the latter evolving into superoxo O^2−^ species (EPR active) upon H_2_ exposure.^[^ ^63^ ^]^ The ability of operando EPR to provide quantitative insights into paramagnetic species, particularly related to metal electronic states, vacancy formation, metal dispersion, and reactive intermediates, enabling the distinction of discrete structures, is desirable and could help advance our understanding of catalyst architectures. Nonetheless, although spectrometers are commercially available, the technique remains largely only practiced by specialized groups, and more needs to be done to improve its accessibility.

Operando IR and, to a lesser extent, Raman spectroscopy studies have mainly been used for studying the formation and evolution of adsorbed reactive intermediates, complementing the insights accessed through NAP‐XPS, but with the advantage of bridging the pressure gap. This requires that the catalyst exhibits sufficient reflectance to perform DRIFTS measurements. Analysis of the DRIFTS spectra with time under steady‐state conditions over an inverse ZrO_2_/Cu catalyst confirm that the reaction likely proceeded via the formate pathway based on the observed vibrations (Figure 12i).^[^ ^35^ ^]^ By switching from steady‐state to CO_2_‐free conditions, it is possible to observe the consumption of the surface species in pure H_2_, showing that carbonate and formate species decrease rapidly, while the accumulation of methoxy species is weak (Figure 12j). Thus, operando DRIFTS can help determine the relative kinetics of different conversion paths.

Although the powerful insights operando spectroscopies can deliver into atomic‐level structural dynamics and their correlation with reactivity patterns, linking this knowledge to catalyst nanostructures remains a significant challenge, especially in complex ternary systems. In this regard, TEM techniques and their associated modalities (e.g., energy dispersive X‐ray (EDX) and electron energy loss (EELS) spectroscopies), particularly in situ measurements, allow the real‐time observation of structural changes at the nanoscale during catalytic reactions. The visualization of single‐phase In_2_O_3_ systems under CO_2_ hydrogenation conditions confirmed the transformation between crystalline and amorphous states (Figure 12k).^[^ ^30^ ^]^ Similarly, in combination with spectroscopy analyses, studies have confirmed the phase distributions in more complex ternary Cu/ZnO/Al_2_O_3_ systems (Figure 12l).^[^ ^35^ ^]^ Only a limited number of in situ TEM studies have been reported in CO_2_ hydrogenation. These have often focused on model systems that are friendly for visualization and, due to challenges associated with the experimental design, few have correlated these insights with kinetic information.^[^ ^35^ ^]^ Nonetheless, the observations highlight their importance for improving understanding of active nanostructures of CO_2_ hydrogenation catalysts and their dynamics.

Technique integration is crucial to obtain a comprehensive view of CO_2_ hydrogenation catalysts under operational conditions. Despite the significant progress, challenges remain in fully exploiting the potential of in situ and operando studies. One major challenge relates to the difficulty in designing experiments that perfectly mimic industrial reaction conditions, which can lead to discrepancies with kinetic data gathered under industrially relevant conditions. Moreover, the complexity of correlating the observed properties with the nanostructure of catalysts, especially in ternary systems where diverse structures may coexist, adds another layer of difficulty. Herein, data science approaches and tools promise to help overcome some current challenges by rapidly analyzing large datasets and uncovering intricate relationships that might not be directly apparent through manual inspection, saving time and resources. For example, recent studies have demonstrated the possibility of identifying hidden property‐performance descriptors through analysis of available literature via big‐data^[^ ^114^ ^]^ or machine‐learning (ML) approaches.^[^ ^115^, ^116^ ^]^ An aspect that needs improvement for mining reported data effectively is the development of standards for describing synthesis, characterization, and performance data, which is currently left to the discretion of authors, reviewers, and publishers.^[^ ^117^ ^]^

Another area of interest is the screening for new catalytic compositions and structures. This can be which has been approached by combining ML with theoretical predictions, for example, of adsorption energies or relevant intermediates.^[^ ^118^, ^119^, ^120^ ^]^ Predictions of active site architectures have also been made through developing microkinetic‐guided machine‐learning pathway searches.^[^ ^85^ ^]^ Herein, a major challenge comprises the synthesizability of predicted structures, which may be highly complex, for example, CuCoNiZnSn‐based high entropy alloys.^[^ ^118^ ^]^ Further, current models for atomistic simulation typically have not addressed the full structural complexity of multi‐component systems. Alternatively, catalyst screening and optimization can be approached using experimental data, potentially combining automation, as recently demonstrated for Cu–Zn–Ce ternary systems.^[^ ^121^ ^]^ In this context, ML‐based methods have yet to identify compositions distinct from the main families already studied. Besides rationalizing and predicting catalytic behavior, we expect ML tools will become routinely used to accelerate, standardize, and advance catalyst characterization. ML‐based methods have already demonstrated strong potential for automated analysis and tracking of the dynamic behavior of catalysts.^[^ ^122^, ^123^, ^124^ ^]^ Generalization across material classes, agreement on standards, and their implementation in user‐friendly software will promote the adoption of these emerging methods.

## 5 Symbiotic Catalytic Interfaces

The compartmentalized approach to developing CO_2_ hydrogenation catalysts, focusing on single catalyst families (Cu‐, In_2_O_3_‐, or ZnZrO*_x_*‐based), has precluded the establishment of general design rules. The overview of developments for single‐, binary‐, or ternary‐phase systems presented in Section 3 has shown that additional phases are introduced to enhance specific properties such as the dispersion of the established active phase, adding redox functionality, or improving interactions that foster oxygen vacancy formation, CO_2_ adsorption capacity, or water desorption.

**Figure 13:**
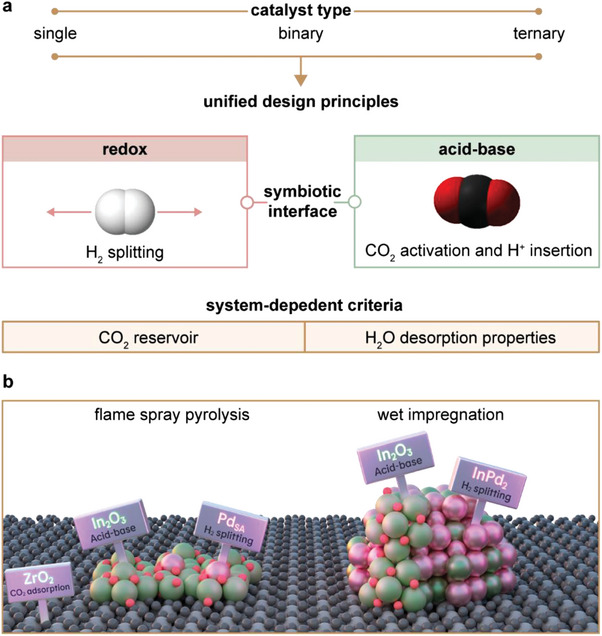
a) Scheme illustrating common design rules with special emphasis on the concept and properties of symbiotic catalytic interfaces reported for catalytic materials for CO_2_ hydrogenation to methanol. b) Key examples of symbiotic catalytic interfaces in ternary Pd‐In_2_O_3_/*m*‐ZrO_2_ catalytic materials prepared by distinct synthesis methods.

A key insight from our analysis is that integrating both acid–base and redox functionalities is crucial for efficient catalyst performance. These functionalities need to be in close proximity, forming what we call a “symbiotic interface” (**Figure** **13**). This interface is essential for efficient H_2_ splitting, CO_2_ activation, and subsequent H_2_ transfer steps, which are critical for the selective hydrogenation process to methanol. The structural realization of this interface varies among different catalyst families, reflecting their unique characteristics and mechanisms. For example, the symbiotic interface in Cu‐based systems involves interactions between Cu and the zinc oxide support. In benchmark Pd‐In_2_O_3_/*m*‐ZrO_2_ catalysts, this can be achieved by integrating highly dispersed and intermixed palladium and indium species on monoclinic ZrO_2_ carriers or by forming Pd–In alloys neighboring In_2_O_3_ patches. Finally, in ZnZrO*_x_* materials, the presence of isolated zinc species on the surface of zirconium particles creates a system with inherent acid–base and redox functionalities. Careful control of phase composition is required to ensure optimal performance.

While the symbiotic interface is a central design criterion for CO_2_ hydrogenation catalysts, other properties have been reported as critical, with the relevance varying between the catalyst families. However, understanding the interplay between the symbiotic interface and these other properties remains limited. One significant challenge in establishing their influence stems from the complexity of the catalyst architectures, particularly in ternary systems where diverse active structures may coexist. Most descriptions of these structures currently rely on density functional theory (DFT) simulations, which may not fully capture the complexities of real systems. Further, these properties are often interdependent, making it difficult to isolate and optimize individual effects. Future research should focus on elucidating the complexities of these interfaces and their impact on catalytic performance, using both experimental and computational approaches.

## 6 Conclusions and Outlook

Although developing CO_2_ hydrogenation catalysts for methanol synthesis has often been tackled for individual types of catalytic material (Cu, In_2_O_3_, or ZnZrO*_x_*‐based), our analysis has identified many similarities in design principles across these systems. In all cases, the intrinsically active phase has been combined with other components, mostly in binary or ternary forms, increasing the catalyst complexity to enhance methanol productivity. These components have generally been categorized as support or promoting phases, but their specific roles do not always fit neatly into a single function, and in many cases, are not yet fully rationalized.

In particular, the active site structures of multicomponent systems may differ from those of single catalytic materials. Our comparison of the different systems shows that proximity between H_2_ splitting and CO_2_ adsorbing and activating sites is a central principle for all catalytic families. We introduce the concept of maximizing symbiotic interfaces in targeted catalyst architectures to enhance activity while preserving selectivity. Interestingly, as recently shown for In_2_O_3_‐based systems, more than one catalyst nanostructure may yield similarly effective performance.

While high stability may be observed across all catalytic families, the structures are known to depend on the reaction conditions. Improving our understanding of the atomic structures that give rise to symbiotic interfaces during operation is currently a key research target. Herein, the correlation of existing operando approaches, and in particular, the further integration of in situ TEM are important goals. The recent adoption of machine‐learning methods has demonstrated utility for accelerating catalyst optimization. With improved chemical knowledge, these models may predict entirely new catalyst compositions and architectures that fulfill the requirements of promising methanol synthesis catalysts. We see them as particularly suited for helping obtain a more precise description of catalytic architectures through combination with analytical techniques. We expect that tackling any of the challenges highlighted will bring exciting advances in catalytic materials design. This approach is not only relevant for methanol synthesis but also for other thermocatalytic CO_2_ conversions.

## Conflict of Interest

The authors declare no conflict of interest.

## Data Availability

All data used in the analysis for Figures 7a–c and 9a–c are available on Zenodo (https://doi.org/10.5281/zenodo.12549958)^[^ ^125^ ^]^ and can also be obtained from the corresponding author.
